# Qualitative analysis of the dynamics of policy design and implementation in hospital funding reform

**DOI:** 10.1371/journal.pone.0191996

**Published:** 2018-01-26

**Authors:** Karen S. Palmer, Adalsteinn D. Brown, Jenna M. Evans, Husayn Marani, Kirstie K. Russell, Danielle Martin, Noah M. Ivers

**Affiliations:** 1 Women’s College Research Institute, Women’s College Hospital, Toronto, Ontario, Canada; 2 Faculty of Health Sciences, Simon Fraser University, Burnaby, British Columbia, Canada; 3 Institute of Health Policy, Management and Evaluation, Dalla Lana School of Public Health, University of Toronto, Toronto, Ontario, Canada; 4 Li Ka Shing Knowledge Institute, St. Michael’s Hospital, Toronto, Ontario, Canada; 5 Enhanced Program Evaluation Unit, Cancer Care Ontario, Toronto, Canada; 6 Institute for Health Systems Solutions and Virtual Care, Women's College Hospital, Toronto, Ontario, Canada; 7 Department of Family and Community Medicine, Women’s College Hospital and University of Toronto, Toronto, Ontario, Canada; Universita degli Studi di Firenze, ITALY

## Abstract

**Background:**

As in many health care systems, some Canadian jurisdictions have begun shifting away from global hospital budgets. Payment for episodes of care has begun to be implemented. Starting in 2012, the Province of Ontario implemented hospital funding reforms comprising three elements: Global Budgets; Health Based Allocation Method (HBAM); and Quality-Based Procedures (QBP). This evaluation focuses on implementation of QBPs, a procedure/diagnosis-specific funding approach involving a pre-set price per episode of care coupled with best practice clinical pathways. We examined whether or not there was consensus in understanding of the program theory underpinning QBPs and how this may have influenced full and effective implementation of this innovative funding model.

**Methods:**

We undertook a formative evaluation of QBP implementation. We used an embedded case study method and in-depth, one-on-one, semi-structured, telephone interviews with key informants at three levels of the health care system: Designers (those who designed the QBP policy); Adoption Supporters (organizations and individuals supporting adoption of QBPs); and Hospital Implementers (those responsible for QBP implementation in hospitals). Thematic analysis involved an inductive approach, incorporating Framework analysis to generate descriptive and explanatory themes that emerged from the data.

**Results:**

Five main findings emerged from our research: (1) Unbeknownst to most key informants, there was neither consistency nor clarity over time among QBP designers in their understanding of the original goal(s) for hospital funding reform; (2) Prior to implementation, the intended hospital funding mechanism transitioned from ABF to QBPs, but most key informants were either unaware of the transition or believe it was intentional; (3) Perception of the primary goal(s) of the policy reform continues to vary within and across all levels of key informants; (4) Four years into implementation, the QBP funding mechanism remains misunderstood; and (5) Ongoing differences in understanding of QBP goals and funding mechanism have created challenges with implementation and difficulties in measuring success.

**Conclusions:**

Policy drift and policy layering affected both the goal and the mechanism of action of hospital funding reform. Lack of early specification in both policy goals and hospital funding mechanism exposed the reform to reactive changes that did not reflect initial intentions. Several challenges further exacerbated implementation of complex hospital funding reforms, including a prolonged implementation schedule, turnover of key staff, and inconsistent messaging over time. These factors altered the trajectory of the hospital funding reforms and created confusion amongst those responsible for implementation. Enacting changes to hospital funding policy through a process that is transparent, collaborative, and intentional may increase the likelihood of achieving intended effects.

## Introduction

As health care systems around the world undergo reform, governments seek to simultaneously control growth in health care spending, improve the patient care experience, and advance population health outcomes–the Triple Aim [1]. One major policy lever for the attainment of such goals is the method of financing health care institutions and providers. Hospital funding reform is thus a common feature of health system restructuring and transformation efforts globally. In its current iteration, hospital funding reform in developed nations often involves concurrent initiatives that attempt to promote accountability through greater transparency [2], improve adherence to evidence-based clinical guidelines [3], expand volume of activity [4], and/or assure equitable access to hospital services [5].

As part of this wave of reforms, some jurisdictions in Canada have recently begun to shift away from global hospital budgets, and instead towards payment for episodes of care based on diagnoses or procedures. For example, Ontario’s Ministry of Health and Long Term Care (MoHLTC) announced that as of April 1, 2011 it would begin a multi-year implementation of “patient-based” payment for hospitals [6], [7]. This occurred within the context of a challenging financial environment that focused a number of policy areas on cost control. The stated goals of the hospital funding reform that subsequently unfolded were aimed at providing “better care for patients at less cost” [8], driving a more sustainable health care system through smarter use of limited resources, and moving away from global budgets (except for small hospitals and forensic mental health services) by tying funding more directly to the quality of care provided to patients [9], [10].

Ultimately, “patient-based” hospital financing was implemented in nearly all Ontario hospitals through two new funding components intended to partially replace global budgets: the Health Based Allocation Method (HBAM), and Quality-Based Procedures (QBP). QBPs were first implemented in Ontario acute care hospitals in April 2012, with a phase-in over time that was intended to allow service providers to anticipate changes and plan for impacts [10]. As there is only one public payer in Ontario for all medically necessary hospital and physician care, and all providers participate in that single funding network, payment policies affect entire classes of hospitals, hospital systems, and health care providers. The only Ontario hospitals excluded from the QBP funding reform were those with unique features that led government to consider them exceptions. Specifically, Ontario’s 55 small hospitals – typically less than 2700 inpatient or day-surgery cases per year in two of the last three years—are excluded from QBP funding component and continue to be funded primarily through global budgets. Likewise, specialty hospitals such as mental health or children’s hospitals, as well as chronic care and rehabilitation hospitals, are also excluded or have only implemented specific QBPs for their patient populations (e.g. tonsillectomy). However, quality elements of the QBP strategy, such as those related to performance management, also apply to small hospitals even though they are excluded from the funding component. Likewise, major investments in standardized order sets linked to QBPs include small hospitals. Practically, this means that small hospitals have had performance management supports around QBPs and some limited expectation of focussing on improving QBPs, but the overwhelming focus of the QBP strategy has been on community and large hospitals.

QBPs are similar in some, but not all, respects to activity-based funding (ABF) reforms [11]. As implemented in Ontario, QBPs involve two components: 1) pre-set reimbursement rates for management of patients with specific diagnoses or who undergo specific procedures (consistent with ABF); and 2) evidence-based, best-practice clinical pathways, incorporated into extensive implementation handbooks, intended to guide the provision of care for each QBP. Financial incentives, coupled with promotion of best-practice pathways, were together intended to reduce variation in clinical practice, so that patients receive “the right care, in the right place, at the right time” [8].

The unique combination of QBP, HBAM, and global budget funding represents a made-in-Ontario solution, the first major change in Ontario’s hospital funding mechanisms since the introduction of global budgets in 1969 [12].

To date, there has been no evaluation of the implementation of QBPs. In this paper, we report on our independent, formative evaluation of QBP implementation, in which we examined whether or not there was consensus in understanding of the program theory [13], [14] underpinning QBPs (i.e. points of agreement and disagreement about how the policy is understood to work in the field vs. how it was intended to work) and how this may have affected implementation. Implementation fidelity is the degree to which an intervention is implemented as intended by the developers [15–17]. Fidelity acts as a mediator of the relationship between interventions and their intended outcomes [15]. In other words, assessment of the fidelity with which QBPs were implemented may help prevent potentially false conclusions from being drawn about their effectiveness.

## Methods

We used an embedded case study method [18] and undertook in-depth, one-on-one, semi-structured, telephone interviews with key informants, each working at one of three levels within the health care system: (Level 1) individuals who conceived of, and were involved in the design of, the QBP policy; (Level 2) individuals in organizations supporting QBP adoption in hospitals across the system; and (Level 3) individuals in hospitals responsible for QBP implementation. An embedded case study allows for in-depth review of new or unclear phenomena while “retaining the holistic and meaningful characteristics of real-life events” [19], as well as for an appreciation of critical elements of context that are essential to understanding policy design and implementation [20]. Case study is one technique consistent with 'realistic evaluation', which aims to find not only which outcomes were produced from interventions but also how they were produced, and in which contextual conditions [21]. We received ethics approval from the Women’s College Research Institute Research Ethics Board.

We also undertook selective document review of both publicly available and proprietary sources (i.e. emails shared with the research team from key informants), which provided background information that was helpful in contextualizing our research [22]. From these documents we abstracted background data on hospital funding reform characteristics (i.e. proportion of intended vs achieved for each of the funding schemes) and on the QBP phase-in schedule. We verified these data with key informants, correcting inaccuracies in publicly available documents when informants provided updated information from more reliable proprietary sources.

### Recruitment

We purposively sampled, seeking information-rich cases while ensuring broad representation, thus allowing for a comparison of commonalities and differences across groups.

For policy designers (Level 1) and adoption supporters (Level 2), we created a list of potential key informants through discussion with research team members and through review of government and Ontario Hospital Association documentation on hospital funding reform and related policies. One team member (AB) directly emailed these individuals to seek their participation and to facilitate communication between them and the interviewer (KP), who subsequently scheduled and conducted telephone interviews with those who agreed to participate.

For hospital implementers (Level 3), we selected hospitals to invite through a multiple-stage, stratified, purposeful, sampling approach, aided by Qualtrics® online survey software. First, we solicited survey input from executives in 14 Local Health Integration Networks (responsible for planning, integrating, and funding local health care), the Ontario Hospital Association, and Cancer Care Ontario (a provincial agency and primary adviser on cancer services, with a province-wide initiative—Access to Care [23], [24]—to monitor and improve wait times for key healthcare services). These respondents were well-positioned to comment on hospital success or lack thereof with respect to QBPs because of their role in system oversight and support. We asked respondents to independently and confidentially select three higher performing hospitals with regard to QBPs and three hospitals with the most room for improvement (lower performing) from among the initial 71 hospitals implementing QBPs, explaining the basis for each selection. We then classified hospitals as follows: within each of the higher/lower performing categories identified we stratified according to academic and community hospitals, and further stratified community hospitals by size (large vs. small) and geographic location (rural vs. urban). We ultimately selected three academic/teaching hospitals (two higher performing; one lower); and two community hospitals (one higher performing; one lower). Together, these five hospitals allowed for rich data and we achieved informational redundancy.

Within hospitals, we initially sought to interview four key hospital employee leadership groups: 1) chief executive (e.g. CEO); 2) financial/decision support (e.g. Chief Financial Officer-CFO); 3) clinical (e.g. VP/ExecutiveVP Clinical Affairs); 4) and medical (e.g. VP Medical Affairs). We pursued snowball sampling beyond these groups where appropriate. We first contacted each hospital CEO to seek permission to include their facility in our research. If they agreed, we asked the CEOs to identify, and connect our team with, key individuals in each hospital leadership group. All interviews were voluntary and confidential.

### Data collection and management

One trained interviewer (KP) conducted all interviews by telephone, following a semi-structured guide to capture information relevant to our research questions. Interviews were audio recorded, professionally transcribed, and de-identified using a unique numerical identifier for each informant, with the data and key code securely stored at Women’s College Research Institute. The interview guide (S1 File) included an introduction, key questions, probing questions, closing questions, and a summary; it was beta tested with key informants and experts and refined prior to commencing the formal interviews. The interviewer made summary case notes immediately after each interview. As key themes were identified during the interviews, they informed the interview guide in an iterative manner so that emerging issues of interest could be pursued. We continued to seek further information at all Levels until researchers agreed that saturation was reached. At Level 3, we sought within-case saturation (i.e. within each case study hospital).

### Analysis approach

Thematic analysis [25] involved an inductive approach, incorporating Framework analysis [26] to generate descriptive and explanatory themes as they emerged from the data. The analysis process included familiarization by immersion in the data, identifying a thematic framework to guide coding, iteratively refining a codebook, indexing and charting the data, and mapping/interpretation [27], [28]. Themes were generated from the data through open (unrestricted) coding to create a template of codes. Two members of the research team (KP, JE) independently open coded two test transcripts to generate themes, then collaboratively reconciled and revised their coding. Using this template of codes, two other members of the research team (HM, KR) then independently coded the first few key informant interview transcripts, comparing and reconciling their coding until they achieved at least 70% agreement on code selection. At that point, duplicate coding was abandoned because of high coherence of coding, but coders consulted with senior members of the research team regularly and whenever coding questions arose. The codebook was iteratively refined throughout the coding process as new concepts became apparent, until no additional codes emerged. Assisted by Quirkos® qualitative data analysis software to facilitate coding, analysis, and data management, we used line-by-line coding and constant comparative methods to understand what was happening with QBPs. Key themes were then independently identified by each of the coders and senior researchers for discussion; upon consensus, data were curated accordingly. Finally, we organized key themes by level of respondent to facilitate comparisons. One researcher (KP) initially identified representative quotations for each key theme, and obtained concensus from NI, AB, JE, and DM. From this we generated meaning by building a logical chain of evidence that was conceptually and theoretically coherent [29]. Validity procedures employed included triangulation to corroborate or refine our findings, seeking disconfirming evidence, researcher reflexivity, peer debriefing, and member checking (i.e. with selected Levels 1 and 2 informants) [30], [31].

## Results

We interviewed 45 key informants between May 18-October 6, 2016, including 12 designers (Level 1), 11 adoption supporters (Level 2), and 22 hospital implementers (Level 3, from 5 case study hospitals). There were no refusals. Interviews ranged from 43–107 minutes in length. In the representative quotes selected here, research team members are designated as “R” and key informant participants as “P”.We also abstracted background information from three key publicly available documents [7], [9], [32].

As described in Table 1, QBPs were phased-in over time, with funds being reallocated from hospitals’ global budgets to their QBP funding envelopes. Starting in FY 2012, for example, four QBPs were introduced, accounting for 6% of the total funding allocation. This was followed in FY 2013 by six more new QBPs, accounting for a combined total of 12% of the hospital funding allocation. Annually since 2012, new QBPs have been phased-in, with commensurate QBP funding incrementally replacing other funding envelopes. Since FY 2014, however, the proportion of actual QBP funding has been half of intended. For example, as of FY 2016 only 15% of hospital funding was allocated for QBPs rather than the intended 30%.

**Table 1:** Ontario hospital funding reform characteristics and phase-in schedule [7], [9], [32].

**Global budget**: fixed annual amount based on historical spending to cover operating expenses, originally intended to comprise 30% of the funding allocation (previously comprised 98.5% of the hospital budget); also includes other targeted funding and post-construction operating costs associated with completion of capital projects						
**Health Based Allocation Model (HBAM):** organizational-level funding based on characteristics of services provided and profile of patients served, originally intended to comprise 40% of the funding allocation (previously comprised 1.5% of the hospital budgets)						
**Quality-based procedures (QBPs):** procedure/diagnosis-specific funding, based on a pre-set price per episode (calculated at the 40th percentile of average costs incurred by all hospitals), coupled with best practice clinical pathways, intended to comprise 30% of the funding allocation (a novel and unique feature of hospital funding reform)						
**Funding Reform**	**FY2011-12** **Pre-QBP**	**FY2012-13** **Phase 1**	**FY2013-14** **Phase 2**	**FY2014-15** **Phase 3**	**FY2015-16**	**FY2016-17**
**QBPs intended** **% funding**	0%	6%	15%	30%	30%	30%
QBPs actual % funding**	0%	6%	12%	13%	14%	15%
**QBPs added by year**	none	primary unilateral hip replacement primary unilateral knee replacement unilateral cataract chronic kidney disease (dialysis)	chronic obstructive pulmonary disease congestive heart failure stroke non-cardiac vascular surgery systemic chemotherapy* gastrointestinal endoscopy*	hip fracture pneumonia tonsillectomy neonatal jaundice bilateral hip and knee replacement	knee arthroscopy cancer surgery (prostate, colorectal)	cancer surgery (breast, thyroid) non-routine and bilateral cataract
**HBAM intended** **% funding**	1.5%	40%	40%	40%	40%	40%
**HBAM actual** % funding**	0%	34%	34%	33%	32%	32%
**Global Budget intended** **% funding**	98.5%	54%	45%	30%	30%	30%
**Global Budget actual** % funding**	100%	60%	54%	54%	54%	54%

* introduced/communicated as QBPs in 2013/14, but funding change took effect in 2014/15

** personal communication, Health System Quality and Funding Division, Ontario MoHLTC

### Main finding 1

Unbeknownst to most key informants, there was neither consistency nor clarity over time among QBP designers in their understanding of the original goal(s) for hospital funding reform.

QBP designers espoused a variety of goals, including improving access, efficiency, transparency, clinical leadership engagement, and reducing variation in cost and/or clinical outcomes. In the absence of commitment to a common goal(s), over time the hospital funding reform policy emphasized a desire to reduce variation in both cost and treatment approach.

#### Designers

“The original objective and intent is really about engaging clinical leadership with best practice and ensuring that clinical leaders within a hospital are engaged with the hospital administration.” Level 1–006

“The goal of QBPs is to use the financial lever to drive quality improvement by establishing really clear prices for different procedures within the hospital setting that correspond with evidence of best practice, with the intent of trying to reduce clinical and practice variation across different hospitals.” Level 1–008

“Ithe goal of QBPs is to use the financial lever to drive quality improvement by establishing realed beyond very high level and broad sort of terms. And they necessarily involve a whole bunch of challenges around communicating them clearly because of their clinical nature.” Level 1–003

### Main finding 2

Prior to implementation, the intended hospital funding mechanism transitioned from ABF to QBPs, but most key informants were either unaware of the transition or believe it was intentional.

The original plan for the reformed hospital funding mechanism in Ontario was a simple ABF approach using a price per category of admission (i.e. diagnosis or procedure), with only a minority of overall hospital funding through a global budget. Instead, by the time the reform was implemented, it had become QBPs, retaining the price per volume formula characteristic of ABF, but coupling that with two other funding mechanisms (global budgets and HBAM), and with an unfunded quality overlay intended to encourage adherence to clinical pathways. This overlay took many forms, from the handbooks and pathways previously described that were intended to have an exhortational or educational impact, to performance reports for some types of QBPs that included a small set of process and/or outcome indicators.

The hospital funding mechanism morphed from a relatively simple funding formula closely aligned with ABF—[(Rate x volume) +/- global budget]—to an elaborate funding formula—[(rate x volume) with a quality overlay] + [global budget] + [HBAM]. The inclusion of a quality overlay appears be have been added to increase the likelihood of clinician engagement in organizational change efforts.

#### Designers

*“*I have been in on it since the beginning. So, I have got a pretty consistent, clear idea of how it started and what the idea is and how we were going to roll this out. But you go and ask people in the field, sometimes it’s a bit of a black box. They don’t always … it hasn’t been really well-communicated and well-transitioned to the field.” Level 1–004

“The idea was let’s expand activity-based funding across basically all activity that we can measure, and the idea then will be everything is now on some sort of a revenue basis, and hospitals then won’t be faced with, well, this procedure doesn’t bring us in any new revenue, so we’ll do less of this. Instead, it’s more a matter of sorting things out according to need, access, and efficiency. That was a key difference between what we proposed around the policy for patient-based payments and what actually ended up happening with QBPs.” Level 1–014

“There was tension between ADMs [Assistant Deputy Ministers] and the people who work with them who favour HBAM and the others who favoured QBPs. So, there was tension between which one works better for a certain period of time.” Level 1–011

#### Adoption Supporters

“…it was always that, first, we’re going to introduce the rate by volume funding and then, we’ll introduce the quality overlay.” Level 2–018

#### Hospital Implementers

“R: Do you know whether other mechanisms than QBPs were considered for achieving the goals that you mentioned? P: I think there was something before that, but I just don’t remember.” Level 3–034

“R: Do you know if they considered other mechanisms to achieve that same goal? P: You know what, I don’t know.” Level 3–040

### Main finding 3

Perception of the primary goal(s) of the policy reform continues to vary within and across all levels of key informants.

Four years after their introduction, there is still a lack of consensus across policy designers, adoption supporters, and hospital implementers and, in particular, among hospital implementers, regarding the specific goals of QBPs and how the hospital funding mechanism is meant to support achievement of these goals. The multifaceted intentions had narrowed to an emphasis on reducing variation in cost and in patient outcomes. However, there remains disagreement about the primary goal. Many adoption supporters and hospital-implementers believe that QBPs are mostly intended to control costs by reducing length of stay; others reported the intent is primarily to enhance quality of patient care by reducing treatment variation. Some key informants feel they are meant to do both, while others question the extent to which these two goals could be simultaneously achieved. Across levels, there was not agreement on whether or not the goals had changed over time.

#### Designers

“I think the goals of the system have changed over time now and are much more focused on the appropriateness or the effectiveness of care. Still somewhat focused on the costs or the costs per unit of care but I’m not sure that access is still the goal of the QBPs. And I’m not sure that the productivity of the system is still the goal of the QBPs, given that the portion of funding that’s allocated to QBPs is lower now than it was originally intended to be.” Level 1–003

“I think the goals have not changed.” Level 1–011

#### Adoption Supporters

“The goal of QBPs is to do a better job of better tying funding to quality.” Level 2–012

“So the real goal was to save money.” Level 2–021

“The stated goal was all about improving the quality of care.” Level 2–018

#### Hospital Implementers

“Is this about quality or is this about saving money? I mean, the theory is that if you standardize care and make care better, costs should fall, but there is, I think, some cynicism in the field about what’s the real goal here. Is the real goal just to save money or is the real goal to improve care?” Level 3–022

“I’ll say the government’s goal is to reduce cost and our goal is to improve patient care.” Level 3–038

“I think the goal is to provide the best patient experience, outcomes, through a standardised process that all the hospitals follow, that’s good for the patient, good for the system, and it’s done economically.” Level 3–027

“I don’t believe they could improve quality of patient care. I believe they’re there to standardize and to reduce costs and consolidate.” Level 3–033

“my gut tells me that the government’s goals changed a little over time. I think that they probably started with a cost-savings or a cost-control … a true need to do that. I get it. And I think that … but to be responsible, well, it has to be quality care. It can’t be just any old care. It has got to meet a minimum standard. It has to be good care. And so, I think they started out with focusing on both sides. But you’re right, the emphasis, I believe, was originally much more on financial than on outcomes for patients. And I feel now that when you approach it from the outcomes for patients, it does change things a little.” Level 3–042

“When we talk to our clinicians, they don’t see the ‘Q’ for quality in QBPs. They think it’s a purely financial focus.” Level 3–025

“I do not consider QBPs a quality metric, to be really, 100% blunt…My only point is, nobody has that conversation, when you’re actually a hospital … when we get our numbers, I don’t hear everybody rushing into my office saying, oh my God, we did so well on QBPs this year, we had such better outcomes of patient experience. I hear, and I hear from my colleagues, oh, how did you do on QBPs, did you lose or did you gain money. Then, it’s not a quality indicator.” Level 3–031

“To be crass, QBPs are really more about funding than they are about quality at this point in their evolution.” Level 3–037

### Main finding 4

Four years into implementation, the QBP funding mechanism remains misunderstood.

Four years into implementation, QBP stakeholders continue to vary in their understanding of the QBP funding mechanism. Some correctly reported that each QBP price is calculated from a pre-specified rate x volume (i.e. ABF) payment scheme; some mistakenly believed each QBP price is based on the actual cost to provide care described in each best-practice pathway; others mistakenly believe payment is contingent on demonstrated adherence to specific quality standards, even though this originally envisioned financial connection between quality and cost was never implemented when QBPs were rolled out. Although policy documents emphasize quality, hospital funding is not tied to patient outcomes or adherence to best practice.

#### Designers

“… we have the means to basically classify all acute inpatient and day surgery activity done in the hospitals. And, because we can do that, we can also assign a price based on average cost to start with, but maybe moving towards introducing these sorts of best-practice prices in certain areas.” Level 1–014

“… take a particular procedure, look at what would be the best quality way in which that procedure could be delivered through the use of an expert panel in terms of that process, and then be able to set a price based on what the best quality could be delivered for that price.” Level 1–004

“So, to be really clear, we don’t price the pathway itself. We just identify the average cost of current service delivery. And if we priced the pathway, we might see something quite different. We might not be able to afford it. (Laughter) I don’t know.” Level 1–006

“the handbook is then priced to determine what is the best practice price associated with the quality or the pathway. R: So, did you say that that pricing occurred based on that clinical pathway? P: That’s right. R: So, to your understanding, the cost of the pathway itself was costed out? P: Yes.” Level 1–007

“it’s very clear that, as a whole, we don’t really understand what health system funding reform and QBP is all about as a whole. And, so, we just leave it to the CFO and kind of trust she holds judgment on that. Most of us don’t even know what the right questions are to ask health management when it comes to these matters.” Level 1–008

#### Adoption Supporters

“the quantity of funds provided for a given procedure were based on the costing of what ideal care would be.” Level 2–015

“at the outset, it was stated that there would be, it was implied or stated, explicitly, that there would be money ties to quality. In fact, there was this sort of famous line, where it said something like payment equals rate times volume plus quality. Obviously, a notional formula, but never was clear how that quality part would be paid and for which QBPs and how, for each QBP. I don’t think it’s really been implemented for any. So, effectively, it’s a rate by volume funding mechanism.” Level 2–018

#### Hospital Implementers

“based on case costing information, we know that doing a hip costs X in the province. And when you take a look at that, they picked the 40th percentile and said, bing, that’s the number and that’s what you’re going to get paid.” Level 3–023

“Thated on case costing information, wcertain goals and initiatives for the QBPs, then you’ll actually be rewarded an amount of money that is distributed. And, I don’t think it’s distributed equally among the hospitals, it’s based on performance. R: So, you understand it more to be a pay-for-performance mechanism, in a sense. P: Certainly paid for better outcomes.” Level 3–029

“I would say that they did not cost quality indicators in the costing. They have quality indicators that you should be tracking as part of a QBP, but to my understanding, they have never used quality indicators to negatively or positively impact a hospital in Ontario. There has been a lot of talk about it, but we have never gone there.” Level 3–025

### Main finding 5

Ongoing differences in understanding of QBP goals and funding mechanism have created challenges with implementation and difficulties in measuring success.

Lack of clarity and consensus on the goals and uncertainty about how the policy will evolve both act as barriers to measuring whether the desired changes in hospitals have been achieved. The tension created by the need to balance multiple goals (cost versus quality; efficiency versus patient experience) was often apparent. However, the pressure to implement, and the explicit link between revenue generation and volume, has focused attention for implementation on finance executives (e.g. CFO), rather than on quality and patient safety managers. This has led to some reported success around shortening LOS to manage cost, but without robust data to assess effects on quality and other outcomes.

#### Designers

“I think there’s an element of simplicity and elegance that was part of the original thinking of just sending a price signal well in advance and letting hospitals have enough time to adjust that feels like is kind of lost somewhere along the way. And QBPs just feels, like I said, complicated for the un-initiated.” Level 1–008

“I think, a significant challenge in the implementation of QBPs, is that there has been no clarity as to what the primary purpose is.” Level 1–045

“It feels like QBP has, over time, attracted more of the attention of the CFO types than the quality types within the hospital setting, for better or for worse. That’s my observation.” Level 1–008

#### Adoption supporters

“What we should have had was a collective think about how to do it. And that did come later, where they started to say, well, what has everybody done and what worked. But it sort of came after the fact.” Level 2–012

“If it’s not fully articulated, it’s this high level that we’re going to tie funding to quality and the whole world will be better but there’s no articulation of how do we actually measure that. The objectives aren’t clear enough for us to know. So to go back to even your earlier question, once it was implemented, I don’t know. I don’t know the measures that are out there to even tell me that some of them have been implemented, other than a process measure that says someone wrote a handbook. Nobody is measuring the quality of delivery according to best practice, so how would we know?” Level 2–021

#### Hospital Implementers

“There’s very little outcome data available to us for anything that we do with patients. That's one of the things I think the whole system grapples with. We have lots of process indicators. We don’t have a lot of outcome indicators.” Level 3–022

“The initial introduction wasn’t done well unfortunately because of the introduction of a 40th percentile arbitrary price point, so that right away made it a cost thing and not a quality thing and the whole thing started to lose a lot of credibility with clinicians and the services really… No one has shown me any information that would say, you know what, we’ve mapped out and costed the perfect care pathway for the CMG, and when you do that, this is what you should have. You should have a length of stay at this. You should have nursing coverage at that. You should have this and that… to provide all the appropriate operational guidance, clinical guidance, to hit the mark that was I think fully intended at the outset.” Level 3–023

“increasingly, money is being taken out of global and being put into HBAM and QBP. And so, it’s an evil funding formula where the total size of the pie doesn’t increase and these are all funding allocation mechanisms as between service providers.” Level 3–024

## Discussion

**Fig 1:**
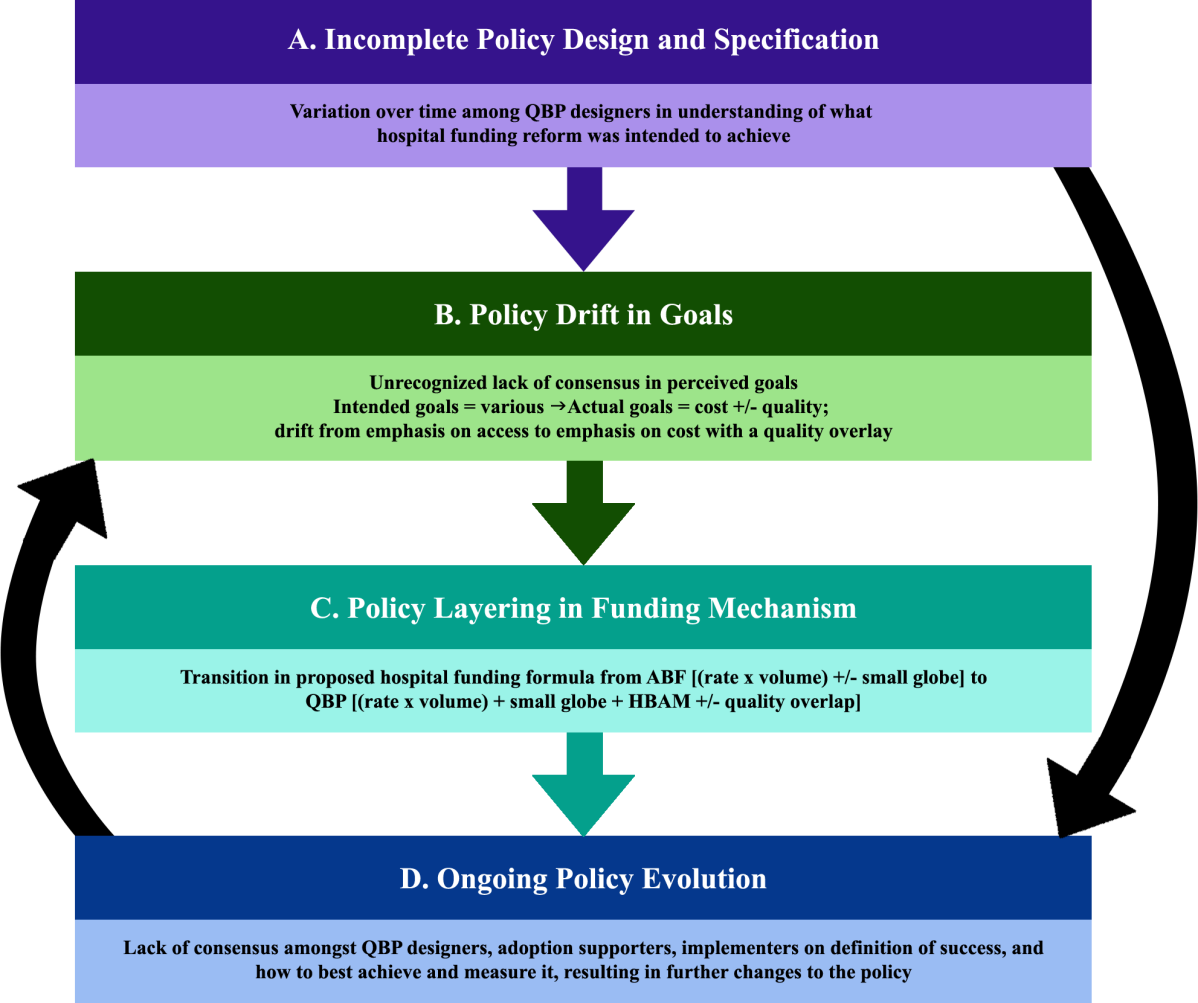
Simplified schematic of principle findings. The evolution of QBP implementation—some purposeful, some unintentional—started with (A) incomplete policy design, which (B) influenced the goals of the policy and (C) changed the funding reform mechanism, ultimately (D) altering the trajectory of the entire hospital funding reform policy.

In Ontario, QBPs represent an important component of hospital funding with the potential for significant impact on hospital policy, care delivery, and budgets. However, as illustrated in Fig 1, several factors adversely affected the implementation of QBPs.

Policy drift is the phenomenon of sometimes oblique transformations [33], [34]. Policy layering refers to the grafting of new elements onto an otherwise stable framework [35], [36]. In QBPs, we see a slowly evolving policy reform, stretched out over many years, with associated variation both in understanding of goals—an example of policy drift—and in the funding reform mechanism—an example of policy layering. Gradual drift in the policy goals, arising from incomplete policy design and specification, changed the intent of the policy; confusing or self-contradictory elements began creeping into the policy text (Main Findings 1, 3, 5). Policy layering altered the mechanism of action—changing it from the originally conceived formula (i.e. rate x volume, plus a small global budget) to the formula that was eventually implemented (i.e. rate x volume, plus HBAM, plus an even smaller global budget)—thereby further shifting the overall trajectory of the reform (Main Findings 2, 4 5). In this way, our research demonstrates how incomplete policy design, incomplete policy specification, and policy drift, together with policy layering, can exacerbate the challenges of implementing funding reform policies in complex adaptive health care systems [37].

What conditions on the ground led to the series of reactions that changed the design, purpose, and construct of the policy [38]? In the case of QBPs, a major transition in funding mechanism was apparently a compromise that evolved organically, rather than by intentional design, with the need for compromise stemming from a lack of consistency in the perceived goals and how best to achieve them. This started with changes in key staff during the design phase, leading to a lack of consistent messaging within the central planning level. The reform goals and funding mechanism were not anchored by a stable, open policy table, meaning there was not a transparent approach to policy design and implementation, involving a consistent cadre of stakeholders who gather regularly, nor a clearly articulated set of policy goals and mechanisms of action to preserve institutional memory [39]. An open policy table could also help to address changes in staffing, a common element of modern public services in which generalist public service leaders are expected to move between roles and to understand all aspects of government [40], but which can limit the duration of expert champions to shepherd reforms. Without consistent champions, weak signals in the communication feedback loop enabled a shift in political attention away from the original goals and mechanism of action. This led to a lack of “stickiness” of the original policy, resulting in policy drift and policy layering. This was likely exacerbated by staff experiencing reform fatigue [41] from too much change, given that QBPs were implemented at the end of a long string of previous health system reforms—31 reforms in all—that began in 2000 [42].

In Ontario, the transition from originally envisioning the implementation of a relatively simple ABF model, to ultimately enacting a more complex hospital funding model, along with ongoing evolution of the policy goals and funding mechanism, has contributed to challenges aligning funding mechanism with policy intent. This, in turn, has made it harder to evaluate or achieve the policy goals. Changes over time, in both the goals and funding reform mechanism, reduced implementation fidelity, suggesting that potential benefits of the original policy goal may not be realized. Indeed, the policy may exacerbate the problem it was intended to fix in the first place. That is, the policy went from a way to protect access to care from the vicissitudes of limited global budgets, to a policy that may reduce access because of volume caps. It also went from a policy that had little intent of reducing costs, to one with a funding mechanism built to constrain costs.

In many cases, government policies should shift over time—consistent with policy learning theory [43]—particularly as the context changes, as new evidence on policy effectiveness emerges, or as the goals of government change. Such shifts should ideally represent purposeful pivots to achieve high-priority goals rather than be based on inertia [44]. These changes, however, can be challenging to the implementation and management of policy in complex systems, in which design, development, support, and implementation are handled at different levels of the health system, and by different actors with varying levels of capacity.

### Relation to prior work

There is scant research on the implementation of QBPs. We know of only one published study evaluating health care leaders’ early responses to implementing QBPs [45]. Prior studies have established the significance of policy drift and layering on social policy reform in, for example, the US [34], [46], Canada [33], [36], Britain/Sweden [47], Chile [48], and other advanced economies [49], [50], and on institutional change in several countries [35]. Our research builds on existing knowledge by applying policy layering and drift (Hacker’s seminal work [34]) to hospital funding reform, demonstrating how these phenomena can be coincident processes. In so doing, we offer new insight into the effects of policy drift and layering on the design and implementation of complex public policy.

### Strengths and limitations

Qualitative research has numerous strengths when properly conducted. We discovered subtleties and complexities—often missed by more positivistic enquiries [51]—about the research topic. However, we acknowledge that qualitative research can be influenced by researcher bias. Our team members made every effort to think reflexively and openly discuss how personal experiences and knowledge could affect the analysis process. We systematically acknowledged preconceived positions, perspectives, assumptions, values, beliefs, and potential biases so they could be discussed and contested [52]. KP is a health policy analyst and researcher who previously led a systematic review of activity-based funding [11]; AB is a public policy researcher, and was previously an Assistant Deputy Minister with the Ontario Ministry of Health and Long Term Care where he worked with key informants through his involvement in health system funding reform; NI and DM are health system researchers and physicians affiliated with an Ontario hospital, not amongst our case study hospitals. We further mitigated the risk of bias through a variety of efforts to ensure validity, as described in the Methods. Another disadvantage of qualitative approaches is that their findings cannot be directly generalized in the same way as quantitative approaches [53]. Sampling in qualitative research is not designed to be representative of wider populations, “but rather purposive to capture a diversity around a phenomenon [26].” We collected data from a sufficient number of key informants to reach saturation on our findings, which fit within, and contribute to, a wider literature on hospital funding reforms.

### Implications of our findings

It is valuable to understand how policies can evolve in complex settings, particularly in health system reform where the goals of policies are often multi-dimensional and reflect both evidence and political compromise. A key challenge is to manage this evolution as effectively as possible. Our findings regarding hospital funding reform may offer lessons in this regard.

In the absence of clear goals, it seems that the QBP funding policy drifted and has become a base onto which many other policies can be layered. It may be that this murkiness served a purpose in the early days of the policy, by destabilizing entrenched ways of doing and by allowing different groups to interpret meaning that they found useful in driving implementation. But if QBPs continue to displace more tranches of global budgets, achieving the goals of hospital funding reform may require greater clarity on policy goals and more transparent, collaborative, and intentional approaches to implementation.

The history of QBPs in Ontario suggests a need to reduce the potential for further confusion amongst stakeholders (agencies, associations, and hospitals) that characterized the early phases of implementation. Despite drift and layering, the current set of Designers have been able to cultivate a strong relationship with Adoption Supporters enabling them to begin to address the issues that arose from drift and layering. An open policy table, with public records of discussion, could support a clear, consistent, and enduring understanding of policy goals and intended mechanisms of action. Broader, more consistent membership at such a table could reinforce fidelity to policy goals and mechanism, and support buy-in to further changes. Creation of a shared repository for minutes, presentations, and policy documents, may enable shared understanding of reforms. The current positive relationships between Designers and Adoption Supporters, and the strong relationships between Adoption Supporters and Hospital Implementers suggest that this tactic could facilitate implementation.

Adoption Supporters and Hospital Implementers have been generally receptive to the quality goals of QBPs. The current approach to calculating the price for QBPs at the 40th percentile of average costs incurred by all hospitals all but guarantees achieving cost-containment goals. Yet, the lack of a direct connection between the clinical care pathway and payment for that care (or measurement of quality) makes the connection between improved patient outcomes and cost unclear. If the primary goal of the hospital funding reform remains two-fold (i.e. reduce costs and improve quality), then a more explicit link between reimbursement and evidence-based clinical pathways might allow the funding reform mechanism to become more aligned with this two-fold policy goal. A more explicit link could help address the tension experienced in the field between the cost and quality goals. However, evidence to date [54], [55] suggests that such highly engineered links may result in unintended negative consequences, or may not achieve desired outcomes without other implementation supports, thus making ongoing evaluation of such links essential. Simultaneously improving quality and reducing costs is most feasible when variation in cost and quality are due to provision of low-value care [56]. Outside of this situation, it may be necessary to have unique system and/or clinical policies for specific, focused goals.

Policies like QBPs that focus on particular procedures or diagnoses could potentially marginalize physicians or hospitals who do not provide QBP procedures or treat patients with QBP diagnoses. There is a risk of systematic preferential attention to QBPs at the expense of non-QBP care—cherry picking—resulting in less managerial attention or fewer quality improvement initiatives for non-QBP care, or preferential selection of QBP patients. This is in line with literature on how performance measurement (and performance management more generally) can lead to a form of myopia or synecdoche (taking a part to stand for a whole) [57]. We found some anecdotal indication of this in our key informant interviews, and are now undertaking quantitative research to evaluate the effects of QBPs on objective indicators such as processes of care, hospital coding behaviour, patient characteristics, and patient outcomes such as length of stay and return to hospital.

## Conclusions

We found that the trajectory of hospital funding reform in Ontario was affected by both policy drift and by policy layering, hazards in any complex system reform. Rather than change occurring by design, the policy reacted organically to changing circumstances on the ground. This shift was enabled by a lack of specification in the goals and mechanism, such that the potential impact of the emergent policy no longer reflected the initial intentions. Enacting changes to hospital funding through a process that is transparent, collaborative, and intentional may increase the likelihood of achieving intended goals.

## Supporting information

S1 File QBP interview guide Nov 24, 2017. (PDF)
