# Correction: Qualitative analysis of the dynamics of policy design and implementation in hospital funding reform

**DOI:** 10.1371/journal.pone.0194280

**Published:** 2018-03-22

**Authors:** Karen S. Palmer, Adalsteinn D. Brown, Jenna M. Evans, Husayn Marani, Kirstie K. Russell, Danielle Martin, Noah M. Ivers

There is an error in the third quotation in the “Designers” subsection under the subheading “Main finding 1” in the Results section. The correct quotation is: “It’s hard to say what the goals of something like government are because they’re very rarely stated beyond very high level and broad sort of terms. And they necessarily involve a whole bunch of challenges around communicating them clearly because of their clinical nature.” Level 1–003

There is an error in the second quotation in the “Hospital Implementers” subsection under the subheading “Main finding 4” in the Results section. The correct quotation is: “That’s my understanding, having met certain goals and initiatives for the QBPs, then you’ll actually be rewarded an amount of money that is distributed. And, I don’t think it’s distributed equally among the hospitals, it’s based on performance. R: So, you understand it more to be a pay-for-performance mechanism, in a sense. P: Certainly paid for better outcomes.” Level 3–029
