# Supplementary material for: Qualitative analysis of the dynamics of policy design and implementation in hospital funding reform
Source: PLoS One. 2018 Jan 26;13(1):e0191996. doi: 10.1371/journal.pone.0191996 (PMC5786323; doi:10.1371/journal.pone.0191996)
Supplement: S1 File — (PDF) [file pone.0191996.s001.pdf]

QBP Semi-Structured Interview Guide

Target Population:
Questions will be administered to three levels of key informants specified in the Protocol:
- Level 1: individuals who conceived of and designed QBPs
- Level 2: individuals in organizations that are supporting QBP adoption
- Level 3: individuals in hospitals responsible for implementing QBPs

Reminder:
no speaker phone

Preamble to Interview:
As part of health system funding reform, the Ontario government introduced a program called Quality-Based Procedures, or QBPs. Through this research, we hope to understand how QBPs are being implemented in Ontario hospitals, how this compares to the expectations of those responsible for developing the QBP policy, what would enable the best-practices described in QBP pathways to be successfully implemented, and what the outcomes of QBPs have been so far. The results will be shared in both publications and presentations.

Consent:
I'll turn on the tape recorder now, read the consent script, and obtain your verbal consent.

Interviewer: Karen Palmer, Strategic Health Policy Lead, WCRI (WCH):

For the purpose of the recording, this is Karen Palmer and I'm interviewing NAME who is the TITLE with ORGANIZATION, a LEVEL (1, 2, 3) key informant, on DATE at TIME (EST).

Thank you so much for agreeing to participate in this study about the development and implementation of Quality Based Procedures (QBPs). Our research is funded by CIHR and the MOHLTC.

Before we proceed with the interview, I'm going to read the consent script to obtain your verbal consent to participate in this study, and to record your responses.

The study does not involve significant risk to you and will take approximately an hour of your time. Your participation is completely voluntary and nobody else, other than those on the research team, will know whether or not you participate. We will not reveal names of institutions, or of individuals interviewed, and interview responses will be non-attributable. The information you provide will be completely de-identified. We may include direct non-attributable quotations in any publications or presentations. Data, including digital recording files and the transcribed interview data, will be appropriately de-identified and securely stored under lock and key at WCRI.

You may decline to answer questions as you see fit, you can ask to stop at any point, and you can request that your responses be excluded from our study data.

By agreeing, you are providing oral consent that you agree to participate in this study, that you agree to audio recording, and that you will answer the questions in this interview to the best of your ability.

If you agree to participate in the study please say, “I agree”.

Participant:

I agree

Interviewer:

Thank you for your oral consent. Now we will proceed with the interview.

Interview Questions:
There are 2 parts to this interview. Part 1 is about QBPs in general, and the implementation process in particular. Part II is about the effects of QBPs.

PART I: These questions are about QBPs in general, and about the implementation process in particular.

A. These questions are about your general understanding of QBPs and health system funding reform (Administered to Level 3 only).

1. What do you understand about QBPs?

   - Probe 1: How would you describe the components of QBPs? (e.g. Have you heard about changes in how we pay hospitals, or the QBP “handbooks” describing clinical pathways, or how money is going to flow according to procedures or numbers of patients?

   - Probe 2: Have you heard about other parts of health system funding reform? If yes, what have you heard?

   - Probe 3: What do you know about how the different parts of health system funding reform work together (if they do work together)?

   - Probe 4: Please describe how your hospital is paid now? How do you see this changing as QBPs, and other aspects of health system reform, roll out at your organization (if appropriate)?

   - Probe 5: Can you describe QBPs with which you have worked in the design, implementation, adoption support, or oversight phase? What was your official and unofficial role in this experience with QBPs?

B. These questions are about the goal of QBPs, the mechanism or chain of events that is supposed to lead to the effect QBPs are supposed to have, and the theory underpinning QBPs.

[Explain if necessary: Underpinning Ontario's new funding program called QBPs is a theoretical framework about why and how QBPs are expected to work. This is called the "program theory". A program theory explains how an intervention — in this case QBPs — is thought to contribute to a chain of results that produce the intended impacts[1]. A program theory explains why, how, and under what conditions the program effects are intended to occur.[2]]

2. **Let's go through those one by one.** (Administered to Levels 1, 2, 3; except Probe 12 to Level 1 only)

Goals (Probes 1-4)

- Probe 1: What's your understanding of the **goal** of QBPs?
- Probe 2: Do you think the **goals** have changed over time?
- Probe 3: If you had to pick one primary **goal** of QBPs, what would it be?
- Probe 4: Are the goals of QBPs, as you perceive them, aligned with your organization's **goals**? (level 3 only)

Mechanisms to achieve goals (Probes 5-9)

- Probe 5: What's your understanding of the **mechanism** by which QBPs are meant to achieve their goals? (i.e. step-by-step flow of how QBPs are supposed to unfold to achieve the goal) Did you do a gap analysis? How long does it take to implement a new QBP, with handbook, end-to-end?
- Probe 6: Do you think there have been changes over time in the **mechanism** of how QBPs are meant to achieve their goals? If so, you've already walked me through how QBPs were supposed to unfold, so can you now walk me through those changes as you understand them? (Don't read this: The intent is to build a flow chart showing respondents perception of QBP evolution.)
- Probe 7: Why were QBPs the policy tool chosen to achieve the goal(s) you mentioned, rather than something else?
- Probe 8: Were options, other than the **mechanism** of QBPs, considered for achieving the goals you mentioned? (If yes: What were they? Why weren't they selected? If no: Why were other options not considered?
- Probe 9: Do you think that people's understanding of the **mechanism** behind QBPs is consistent across QBPs? Why or why not? Is it consistent across organizations? Why or why not?

Theory (i.e. the system of ideas and evidence supporting a belief that the goal can be achieved by that mechanism?) (Probes 10-11)

- Probe 10: What's your understanding of the **theory** underpinning QBPs?

- Probe 11: Do you know of, or believe there to be, evidence underlying the **theory** for QBPs? If yes, what do you feel is the level, the quality, and the relevance of the evidence supporting the program theory for QBPs?

Tools/techniques (Probe 12)

- Probe 12: Did any process improvement techniques or tools inform the design of QBPs? (such as Lean, Six Sigma, CQI, or other quality improvement approaches) (Level 1 only)

C. These questions are about the extent to which QBPs are actually being implemented.

3. To what degree are QBPs being implemented? (Administered to Levels 1, 2, 3; Only Probe 2 to Levels 1, 2)

   - Probe 1: Which QBPs are active in your hospital right now? (Level 3 only)

   - Probe 2: How do you think other organizations in the health system (LHINs, hospitals, the Ministry) view what's happening in terms of QBP implementation? Do you think that organizations elsewhere have a similar appreciation of the state of implementation? (Levels 1, 2 only)

4. What do you think **successful implementation of QBPs looks like** for organizations? (July 5 2016: "would look like") (Administered to Levels 1, 2, 3)

   - Probe 1: How do you think the implementation is going (within your organization, Levels 2, 3) compared to the intended or anticipated path?

   - Probe 2: Do you think there is a shared impression of how implementation is going (stop here for Level 1) within your unit or institution or agency or division?

   - Probe 3: Are all of the QBPs at similar stages of implementation? Why or why not?

   - Probe 4: Are some QBPs easier to implement than others? If yes, why do you think that is?

   - Probe 5: Were there specific QBPs that helped or hindered implementation of other QBPs?

   - Probe 6: Are there specific critical learnings from specific QBPs? If so, what are they?

5. In terms of **fidelity to the original design and original mechanism for implementing QBPs**, in your experience to date, do you think that QBPs are being implemented in the way you understand they were designed? (Please explain) (Administered to Levels 1, 2, 3)

    - Probe 1: If it is not, is the gap in how QBPs are being implemented or is it in their design? If it's a gap in how they are being implemented, where is that gap (e.g. policy level, funder, LHIN, hospital management, unit level leadership)? If it's a problem in the design of QBPs themselves, what's the problem?

    - Probe 2: If not, was there ever a time when there was fidelity, with QBPs being implemented as they were originally designed, and if so, what led to shift away from that?

D. These questions are about **accountability for implementing QBPs**.

6. What is your understanding of your own, and your organization's, **accountability** for the successful implementation of QBPs? (July 6, 2016: who is accountable for what, to whom?) Administered to Levels 1, 2, 3)

    - Probe 1: To whom is your organization accountable for the successful implementation of QBPs?

    - Probe 2: At the personal level, to whom are you personally accountable for QBP implementation, and how do they measure your success in implementing QBPs?

    - Probe 3: At the organizational level, how is QBP implementation being measured, or assessed, or evaluated?

7. Let's talk about the **major barriers and facilitators, in terms of people and personalities,** to the successful implementation of QBPs. (Administered to Levels 1, 2, 3, except as noted)

    - Probe 1: Speaking generally, how does **organizational culture** affect the successful implementation of QBPs? (Level 1 stop here) In your organization? Elsewhere in the system?

    - Probe 2: Speaking generally, how does **organizational leadership** affect the success of QBPs? (Level 1 stop here) In your organization? Elsewhere in the system?

    - Probe 3: Do you think different players in an organization have different **tolerance for QBPs** (e.g. administrators, clinicians)?

    - Probe 4: How does your own **personal capacity** to manage change and analyze/improve performance affect the success of QBPs? (Levels 3 only)

- Probe 5: Have the barriers and facilitators to success varied depending on the QBP?

8. What **supports or enablers, in terms of tools and data,** been important to the uptake or effectiveness of QBPs? (Administered to Levels 1, 2, 3)

    - Probe 1: Do you think any specific supports or enablers would help improve the speed, efficiency, effectiveness, and/or impact of QBP implementation, and why would they help?

    - Probe 2: Have those supports or enablers been suggested at your organization? If so, have they been implemented? If not, why not?

    - Probe 3: Who in your organization is likely to be most affected by the changes that will come as a result of QBPs? How likely are they to change their behavior to adapt to QBPs? How can they be encouraged to change? (Levels 2, 3)

9. I'm going to read you a list of adoption tools and supports. Which of these **tools and supports** would help to enable QBP adoption, and why do you think they would help? (Answer yes/no to each, and whether it would help if these were developed and delivered centrally (say by HQO, or another adoption agency, or provincially, or by the LHIN) or locally (by each provider organization or hospital). Who should develop and deploy these tools/supports? (Administered to Levels 1, 2, 3).

[Explain: Read HQO list of interventions in right column]

Table 1. Mapping Adoption Tools and Supports to Terminology from the Implementation Science Research Literature

Proposed Adoption Tools and Supports	Implementation Science Literature Terminology
Baseline data<br>Ongoing/real-time<br>Audit and Feedback<br>Goal setting/benchmarking<br>Evaluation	Audit and Feedback
Data support<br>Standardized order-set templates	Clinical decision support systems such as:<br>Reminders<br>Order-sets<br>Checklists
Coaching/SWAT team	Practice Facilitation (somebody who comes to coach the local team, or ward, on for example work flow, or QI coaching on a specific problem.)<br>Educational outreach<br>Academic detailing

	Mentoring
Provincial/regional experts	Opinion leaders
Provincial conferences/webinars	Continuing education through online education or conferences
Community of Practice	Community of Practice<br>Learning Collaborative<br>Quality Improvement Collaborative
Best practice recommendations<br>Summary pathway<br>Implementation checklist	Printed educational materials<br>Guidance documents (including Clinical practice guidelines and Clinical/Care pathways)
Clinician engagement strategy	Clinician/Physician engagement strategies, such as<br>Clinical champions/Opinion leaders/Change agents
Recommendations for local/provincial infrastructure<br>Data support for forecasting/budgeting	Organizational context (analytical capacity support for operational or clinical data

10. Is there **anything else** you would like to tell me about the QBP implementation process? (Administered to Levels 1, 2, 3)

PART II: We're almost done. These questions are about the *effects of QBPs themselves after they've been implemented.*

1. What are some of the **effects to date of the QBPs** now implemented? (Administered to Levels 1, 2, 3, except new Probes 6 and 7 to level 3 only)
    - Probe 1: Do you collect or review data that measure whether QBPs are successful in obtaining their desired effect? If yes, which outcomes do you measure (e.g. quality indicators, cost, patient outcomes)?
    - Probe 2: Do you think QBPs are having any impact whatsoever? If not, why not? If yes, describe that impact. To what extent are you measuring adherence to the pathway?
    - Probe 3: What do you think is really responsible for the effect? QBP funding reform? QBP clinical pathways/handbooks to guide quality? Other QBP adoptions supports? Something else? Why do you think that's driving change?
    - Probe 4: Do you feel QBPs are achieving their intended goals?
    - Probe 5: Knowing what you know now, do you think QBPs was the right policy tool to achieve the intended goals? If not, or even if so, what course correction would you suggest?
    - Probe 6: Do you know whether or not the costs to provide care for each QBP in your hospital are consistent with the price you are paid by the Ministry for each QBP? (i.e. Are the Ministry prices assigned to each QBP higher/lower/same than/as your hospital's costs to deliver care for each QBP?) If different, why do you feel your costs are different from those

assigned by the Ministry to each QBP? How do you know what your cost per weighted case is?

- Probe 7: If gains or losses: What are you doing with the financial gains/surplus? How are you handling the losses/insufficient funds?

2. Let's talk about intended and unintended consequences of QBPs.

   - Probe 1: Have there been unintended consequences of the implementation? If so, have these been recognized by organizational leadership? If not, why not?

   - Probe 2: Do you think that some hospitals are getting out of certain lines of work is an intended consequence of QBPs? Do you think that's a desirable or not desirable outcome? Has QBP driven consolidation?

   - Probe 3: Has your day-to-day work or your work load, or that of your colleagues, changed as a result of QBPs? If so, how? What types of work have increased (e.g. administrative)?

   - Probe 4: Are there other unintended or intended consequences?

3. Can we follow up with you if we need to clarify any points you have made during this interview? (Administered to Levels 1, 2, 3)

   - Probe 2: Do you have names of people who would be valuable to speak with? Who was involved in implementing a handbook? If so, do agree to allow your name to be revealed to subsequent potential participants whom you recommend we contact?

4. This is the last question. Is there anything else you'd like to discuss?

5. Thank you very much for your participation. For recording purposes, this is the end of the interview and I'm turning off the recorder now. END

---

[1] http://betterevaluation.org/plan/define/develop_logic_model

[2] Sidani, S., & Sechrest, L Putting program theory into operation. American Journal of

[2] Sidani, S., & Sechrest, L Putting program theory into operation. American Journal of Evaluation, 20(2), 227-238, (1999).
